# Audience Interbrain Synchrony During Live Music Is Shaped by Both the Number of People Sharing Pleasure and the Strength of This Pleasure

**DOI:** 10.3389/fnhum.2022.855778

**Published:** 2022-05-06

**Authors:** Thibault Chabin, Damien Gabriel, Alexandre Comte, Lionel Pazart

**Affiliations:** ^1^Centre Hospitalier Universitaire de Besançon, Centre d'Investigation Clinique INSERM CIC 1431, Besançon, France; ^2^Plateforme de Neuroimagerie Fonctionnelle et Neurostimulation Neuraxess, Centre Hospitalier Universitaire de Besançon, Université de Bourgogne Franche-Comté, Besançon, France; ^3^Laboratoire de Recherches Intégratives en Neurosciences et Psychologie Cognitive, Université Bourgogne Franche-Comté, Besançon, France

**Keywords:** EEG hyperscanning, cerebral coupling, emotional connection, emotional sharing, live performance, musical reward

## Abstract

The study of interbrain coupling in a group of people attending a concert together is a favorable framework to estimate group emotions and more precisely emotional connection between people sharing situations in the same environment. It offers the advantage of studying interactions at the group level. By recording the cerebral activity of people from an audience during a concert using electroencephalography, we previously demonstrated that the higher the emotions and the physically closer the people were, the more the interbrain synchrony (IBS) was enhanced. To further investigate the parameters that shaped inter-brain synchronization in this context, we now focus on the emotional dynamics of the group as a whole by identifying specific moments in the concert that evoked strong or weak emotions, as well as strong or weak emotional cohesion between individuals. We demonstrated that audience interbrain synchrony is mainly associated with experiencing high musical pleasure and that the group emotional cohesion can enhance IBS, but alone is not the major parameter that shapes it in this context.

## 1. Introduction

The study of cerebral coupling during social interactions through hyperscanning paradigms has shifted in recent years, with a huge volume of original research in various controlled (Leong et al., 2017; Mller et al., 2021) or more ecological settings and natural environments (Dikker et al., 2017; Chabin et al., 2021; Reinero et al., 2021). Although very challenging, natural environments offer the opportunity to further study direct (conversation, cooperation, etc.) as well as indirect (when people simply share a situation without explicit verbal or nonverbal communication) social interactions without simulating an experimental setting. Performing measurements in these conditions makes the environment naturalistic. It promotes participants' immersion in the experiment (Czepiel et al., 2021; Wald-Fuhrmann et al., 2021) and natural interactions with both environments and people. Interbrain synchrony (IBS) can be conceptualized “as a neural mechanism that causally facilitates social interaction”; supporting the hypothesis that the natural neural synchrony enhances social interaction, or conceptualized as the simple a consequence of neural entrainment elicited when sharing the same sensory inputs (Novembre and Iannetti, 2021). While is it still open to debate, recent data from hyperscanning studies suggest that IBS is not only shaped by simultaneous integration of similar external inputs (Novembre and Iannetti, 2021), but may have the potential to support social interactions. In brief, being in sync with peers could potentially modify the way we interact. For example, social interactions in rodents can be mediated by simultaneously synchronizing or desynchronizing populations of neurons in the brains of two individuals using intra-brain light stimulations (Yang et al., 2021). Also, visual cues have been reported to influence IBS when performing a face to face baseline before shared appraisal situations (Dikker et al., 2017) and during direct social interactions (Leong et al., 2017) in humans.

To investigate whether positive musical emotions, referred to as pleasure, can be shared between people even in indirect interactions (Chabin et al., 2021), our previous research aimed to estimate emotional neurophysiological synchronization by recording the brain activity of people attending a concert together using electroencephalography, while they were reporting their emotions in real-time (four levels to be reported from neutral = 1, low pleasure = 2, and high pleasure = 3 up to “musical chills” = 4). In the traditional frame of classical concerts, people are expected to minimize overt interactions toward others and are not expected to explicitly influence their peers' behavior (Tschacher et al., 2021). Nevertheless, we mainly demonstrated that IBS was enhanced when people felt similar and strong emotions, but also that the social context can influence IBS even in case of indirect communication. The closer the people were physically, the more similar their emotional reports were. More importantly, the closer they were, the higher was the cerebral synchrony (as well as the physiological synchrony measured using electrodermal activity) when they were reporting high levels of pleasure related to the music.

While our findings are correlational in nature, we defend the idea that sharing similar external inputs is not the only factor that explains these findings. We hypothesized that sharing a high level of pleasure related to music at the same time naturally elicits similar brain activity for several participants, thus enhancing IBS. Further, considering the relationship between physical proximity and IBS (Chabin et al., 2021), we propose that direct non-verbal communication elicited by the desire to share a pleasant emotion with others reinforces or sustains this effect through a retroactive loop. Reciprocally, we recently considered an alternative hypothesis that suggests that interbrain synchrony can shape social interaction to a certain extent, we can also consider that the specific cortical activities (in the frontotemporal areas in the theta frequency band Chabin et al., 2020) related to musical reward processing, shape IBS and enhance a natural implicit form of social interaction, in particular with peers that are physically close.

Since our design overcome the simple dyadic interaction, usually met in hyperscanning paradigms, we are now able to focus the analyses on the emotional dynamic of the overall group. Thus, we still wonder whether the global group emotional cohesion (GEC)–defined here by the reporting of the same emotions at the same time in a majority of people from the sample (*n* = 15), independently of the strength of emotion–was sufficient to elicit enhanced IBS, or concomitantly, whether the strength of emotions alone drove it. In other words, is IBS associated in the first place with being “on the same wavelength” as a group, and potentially in an unconscious way by perceiving a certain emotional closeness/connection with people? If yes, these results could argue for the social effect of IBS theory. This report aims to shed light on the way the emotion of the group plays a role in the global group IBS and, to a certain extent, estimate how the experiment is more than an aesthetic individual experience but also “a communal experience” (Tröndle, 2020) in the context of a concert audience. We expected that both the emotional cohesion and the strength of emotion would shape the IBS; the more the emotion and the number of people sharing it will be high, the more IBS should be high as well.

## 2. Methods

Only a brief method section is provided here. Full details about procedures, recruitment, method, data processing, and analysis can be found in the methods section of our previous publication (Chabin et al., 2021).

### 2.1. Participants

Our participants were recruited among people who had bought a ticket for the semi final round of the International Competition for Young Conductors held in Besanon, France (september 2019). Our sample was composed of 15 participants (12 women) with a mean age of 55.7 years (SD = 18.9, range = 18–78; seven were musicians, including one professional). The study met the local ethical regulations as laid out in French law concerning non-invasive protocols involving healthy participants and was classified as an observational study outside the scope of the Jard law (Article R1121-1 of the French Law Code of Public Health amended by decree n 2017–884 of May 2017). It was submitted to the Ethics Committee CPP Est II, which exempted the study from the full ethics review process. Each participant was informed of the observational nature of the study and signed a non opposition notice designed for observational studies. The participants received no compensation for participation in the study.

### 2.2. Procedure

Participants were seated on the same row of seats, at the first balcony of the concert room. They were only instructed to enjoy the concert, to stay calm and quiet and to limit unnecessary movements. We used a unilateral emotional scale that was a gradient of positive pleasure. Participants reported in real time the pleasure related to music on a smartphone according to 4 levels of positive pleasure (neutral, low pleasure and high pleasure up to chill). They listened to 6 professional conductors who conducted in turn a full symphonic orchestra and choir, performing several extracts of Francis Poulenc Stabat Mater. Twelve of the 15 participants were equipped with consumer-grade EEG headset.

### 2.3. Estimation of Interbrain Synchrony

We distinguished two main components for the estimation of IBS that were; group emotional cohesion (GEC) and group emotional state (GES).

**Figure 1:**
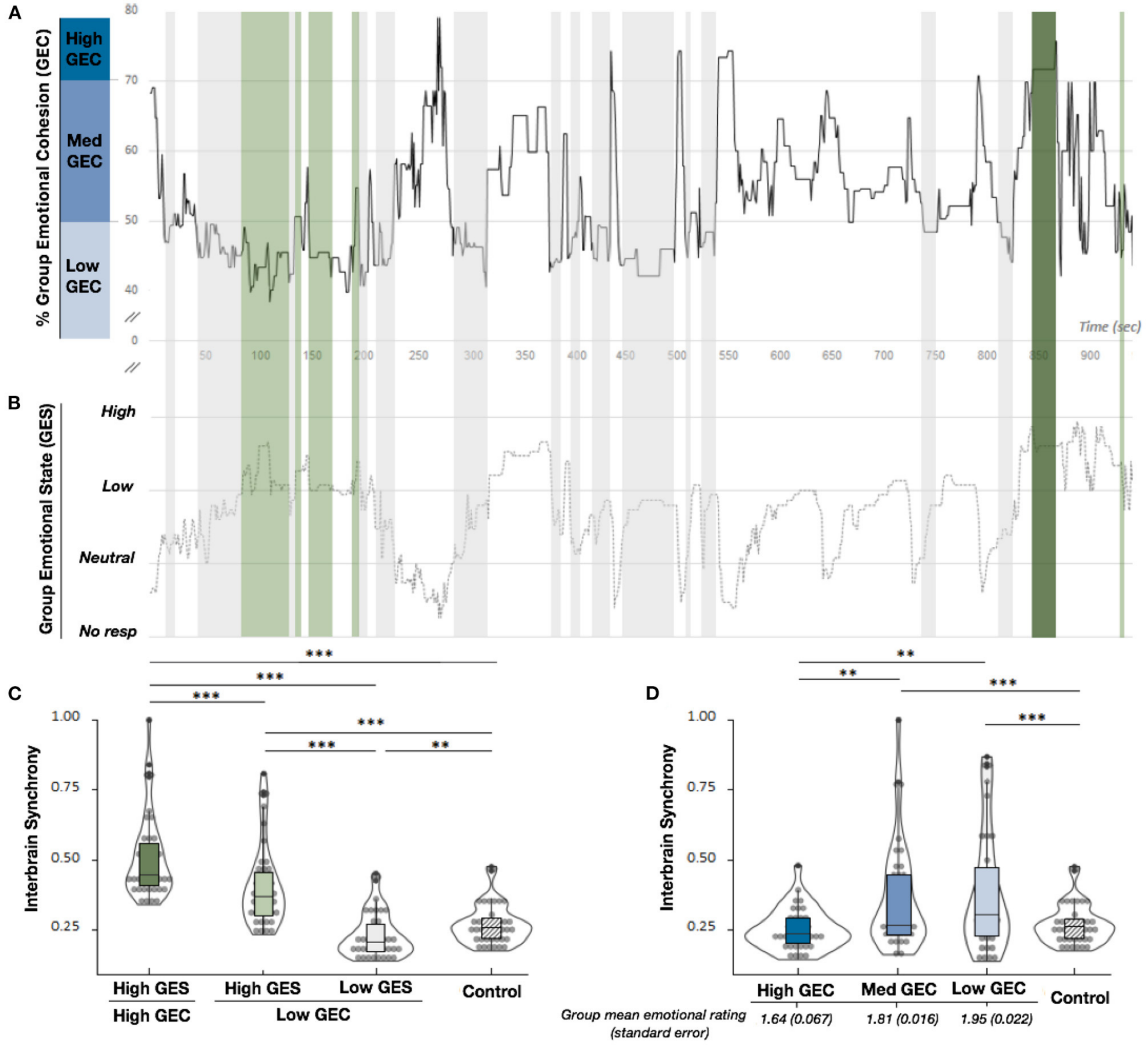
**(A)** Representation of the averaged percentage of group emotional cohesion (GEC) (*n* = 15 giving 105 pairs) derived from behavioral reports for one conductor performance calculated at a temporal precision to the nearest second. This is only a representation, analyses have been conducted with data collected throughout the concert. High GEC is in navy blue **(A)**, Med GEC is in cobalt blue **(A)** and Low GEC is in sky blue **(A)**. **(B)** Representation of the average of the group pleasure reports (n = 105 pairs) on smartphones according to four levels; Neutral, Low pleasure and High pleasure up to chills. Colors correspond to a specific moment visible on the schematic representation of both group emotional state and group emotional cohesion; High GEC/High GES in dark green, Low GEC/High GES in light green, and Low GEC/Low GES in light gray. **(C)** Box plot of the theta coherence estimation calculated during High vs. Low group emotional cohesion for high group emotional state or low group emotional state related to music [see **(A,B)**, green/gray]. **(D)** Box plot of the theta coherence estimation calculated when the group had a High GEC [navy blue **(A)**], a moderate GEC [cobalt blue **(A)**], or a low GEC [sky blue **(A)**]. For both plots: ^**^p < 0.01, and ^***^p < 0.001.

-***Group Emotional Cohesion*** informs about whether the participants were emotionally aligned during the concert. When two participants reported the same level of pleasure, the value is 100%, 50% when participants reported levels of pleasure that differ by 1 point (e.g., high vs. low pleasure), 25% when participants reported levels of pleasure that differ by 2 points, and 0% for differences of more than 2 points. The final GEC index is an average of all 105 pairs GEC index, calculated at a temporal precision to the nearest second. We separated GEC in 3 sub components; High GEC corresponds to group synchrony higher than 70% (navy blue Figure 1A), Med GEC corresponds to group synchrony between 50 and 70% (cobalt blue Figure 1A), while Low GEC corresponds to synchrony lower than 50% (sky blue Figure 1A). A representation of GEC is given on the Figure 1A.

-***Group Emotional State*** represents the strength of the group pleasure related to music and is calculated by averaging the group emotional responses with a temporal precision to the nearest second. We separated GES in two subcomponents; High GES when the overall group reported a degree of pleasure higher than the low pleasure level and Low GES when the group reported a degree of pleasure lower than the low pleasure. A representation of the GES is given on the Figure 1B.

-***Theta***-**Interbrain Synchrony:** EEG data have been recorded with two references on parietal sites (P3 (CMS) and P4 (DRL) with EMOTIV^®^ EPOC+. The overall method for IBS estimation is a replication of the method of Dikker et al. (2017) and Chabin et al. (2021). In brief, EEG data were low- and high-pass filtered (Butterworth) between 1 and 30 Hz, with a notch filter fixed to 50 Hz using the Cartool software. We performed a principal component analysis (PCA) to avoid any rhythmical eye blink influence over IBS. Then we computed IBS defined as θ-IBS by performing a coherence calculation between the signals acquired on similar electrodes for two participants (over frontal, prefrontal and temporal electrodes; AF3, AF4, F3, F4, F7, F8, FC5, FC6, T7, T8 electrodes) using power spectral density (4–8 Hz). It informs about “the amount of mutual information between the two systems,” with a value of 0 when signals are independent and 1 when there is a strong linear relationship between signals. The overall IBS index is an average of IBS estimated for each pair of electrodes for all pairs of participants. Phase and antiphase signals do not influence the result of the calculation. θ-IBS has been computed for High GES/High GEC (when a great number of people of the group shared a high emotional state related to music), for High GES/Low GEC (when a low number of people of the group shared a high emotional state related to music), for Low GES/Low GEC (when a low number of people of the group shared a low emotional state related to music). The low number of events and the minimum number of epochs free from artifacts required to include pairs did not allow us to calculate the condition High GEC/Low GES. We also computed θ-IBS when people from the group were highly (High GEC), moderately (Med GEC), or lowly (Low GEC) aligned in terms of emotional reports independently of the GES. Finally, the control condition corresponds to a random selection of music time periods. The number of epochs selected for the control condition was similar to the amount of epochs got in other conditions.

## 3. Results and Discussion

As expected, θ-IBS estimation revealed that theta coherence was shaped by both GEC and GES, since our results revealed significant differences between each condition (Friedman test χ^2^_(2)_ = 79.4, *P* < 0.0001, Kendall's *W* = 0.68; for *n* = 39 pairs with enough common periods of signal free from artifacts, see Figure 1C). IBS was significantly higher when both GEC and GES were high, and this effect progressively decreased when the GEC decreased but the GES still high, and even more when both the GEC and GES were low (*p* < 0.001 in each case; Durbin Conover *post-hoc* test). As a control, we also estimated θ-IBS for High GEC, Med GEC or Low GEC only, independently of the strength of group emotion [Friedman test χ^2^_(3)_ = 27.1, *P* < 0.0001, Kendall's *W* = 0.25; for *n* = 36] and counterintuitively, simple emotional alignment did not increase θ-IBS. *Post-hoc* tests show a higher theta coherence for Low and Med GEC compared to High GEC (Figure 1D). We imputed the low θ-IBS for High GEC (compared to Med and Low Sync, *p* < 0.01) to the lower emotional ratings reported by the group (mean 1.64, SD = 0.067) that potentially influenced θ-IBS estimation.

This new set of results suggests that, in the context of the concert, audience interbrain synchrony is mainly associated with experiencing high musical pleasure. Considering our previous results which suggest that the IBS was higher when people share intense emotions (Chabin et al., 2021), mixed with the results presented on the Figure 1D, we suggest that the group emotional cohesion can enhance θ-IBS, but alone is not the major parameter that shapes it in this context. These findings also resonate with other recent studies that focused on group's collective physiological responses in the context of the concert. For example, Ardizzi et al. (2020) demonstrated that cardiac synchrony was spontaneously elicited by both the shared stimulations and shared/coherent explicit emotional experience. Czepiel et al. (2021) also demonstrated that specific moment of the concert linked with variations of musical characteristics and producing variations in arousal, engagement and familiarity elicited higher physiological synchrony and finally (Tschacher et al., 2021) suggested that “synchrony may yield an objective signature of aesthetic immersion at the collective level of a concert audience.” We defend the idea that simply shared stimulation should not be the main component driving the θ-IBS here, while we recognize that it can play a role as well. Selected periods for our analyses were only based on emotional reports provided by participants, thus collective auditory/sensory inputs cannot be considered as the root cause producing the main effect. The control condition, through which auditory and sensory stimulations were the same for all participants as in other conditions, is significantly lower than both High GES conditions, thus providing a supplementary argument in favor of this hypothesis.

This exploratory work is a first step toward the investigation of a group emotional dynamic and aims to bring complementary results for our previous publication (Chabin et al., 2021). Several recent works also demonstrated that a perceiving a significant interaction or shared intentionality leads to exhibited marked IBS (Provolovski and Perlmutter, 2021). Here we seek to explore whether minimal interactions produce the same effect. We now wonder to which extent sharing high levels of emotions that elicit a higher θ-IBS, shaped the group emotional dynamic. In this case, interventional research studies should now investigate more deeply the social interactions at the group level to highlight not only correlational, but also causal evidence for IBS (Novembre and Iannetti, 2021).

## Data Availability Statement

The datasets generated for this study can be found in a figshare repository: https://doi.org/10.6084/m9.figshare.14401337.

## Ethics Statement

Ethical review and approval was not required for the study on human participants in accordance with the local legislation and institutional requirements. The participants signed a non opposition notice designed for observational studies.

## Author Contributions

TC: conceptualization, data acquisition, formal analysis, writing original draft, writing—review, and editing. DG: conceptualization, data acquisition, formal analysis, and writing—review. LP and AC: conceptualization, data acquisition, and writing—review. All authors contributed to the article and approved the submitted version.

## Funding

This study was carried out from own funds of the CIC-IT 1431 and the platform Neuraxess, with the material and the human support from the Integrative and Clinical Neuroscience Laboratory of Besançon. The study also benefited from a doctoral grant from the French Ministry of Higher Education and Research.

## Conflict of Interest

The authors declare that the research was conducted in the absence of any commercial or financial relationships that could be construed as a potential conflict of interest.

## Publisher's Note

All claims expressed in this article are solely those of the authors and do not necessarily represent those of their affiliated organizations, or those of the publisher, the editors and the reviewers. Any product that may be evaluated in this article, or claim that may be made by its manufacturer, is not guaranteed or endorsed by the publisher.
